# Standardized procedure prevents perioperative and early complications in totally implantable venous-access ports—a complication analysis of more than 1000 TIVAP implantations

**DOI:** 10.1007/s00423-022-02656-9

**Published:** 2022-09-07

**Authors:** Karolin Thiel, Sarah Kalmbach, Gerhard Maier, Dörte Wichmann, Martin Schenk, Alfred Königsrainer, Christian Thiel

**Affiliations:** Department of General, Visceral and Transplant Surgery, Tuebingen University Hospital, Hoppe-Seyler-Strasse 3, 72076 Tuebingen, Germany

**Keywords:** Totally implantable venous-access ports, Port catheter implantation, Complication analysis, Port catheter explantation

## Abstract

**Purpose:**

Since their invention 40 years ago, totally implantable venous-access ports (TIVAPs) have become indispensable in cancer treatment. The aim of our study was to analyze complications under standardized operative and perioperative procedures and to identify risk factors for premature port catheter explantation.

**Methods:**

A total of 1008 consecutive TIVAP implantations were studied for success rate, perioperative, early, and late complications. Surgical, clinical, and demographic factors were analyzed as potential risk factors for emergency port catheter explantation.

**Results:**

Successful surgical TIVAP implantation was achieved in 1005/1008 (99.7%) cases. No intraoperative or perioperative complications occurred. A total of 32 early complications and 88 late complications were observed leading to explantation in 11/32 (34.4%) and 34/88 (38.6%) cases, respectively. The most common complications were infections in 4.7% followed by thrombosis in 3.6%. Parameters that correlated with unplanned TIVAP explantation were gender (port in situ: female 95% vs. male 91%, *p* = 0.01), underlying disease (breast cancer 97% vs. gastrointestinal 89%, *p* = 0.004), indication (chemotherapy 95% vs. combination of chemotherapy and parenteral nutrition 64%, *p* < 0.0001), and type of complication (infection 13.4% vs. TIVAP-related complication 54% and thrombosis 95%, *p* < 0.0001).

**Conclusion:**

Standardized operative and perioperative TIVAP implantation procedures provide excellent results and low explantation rate.

## Introduction

Forty years ago, the first totally implantable venous-access ports (TIVAPs) were developed by Niederhuber and meanwhile TIVAPs are essential in cancer treatment [1]. The rate of implanted TIVAPs is still constantly rising because of the increasing incidence of oncological malignancies and the development of new multimodal therapy regimens. These ports permit safe long-term administration of chemotherapeutic agents, parenteral nutrition, and antimicrobial treatment [2]. Because of these versatile applications, they are suitable not only in solid-tumor cancers and hematological malignancies, but also in chronic disease such as cystic fibrosis and HIV [3, 4].

Despite the fact that TIVAPs offer many advantages like reliability and safety, especially for cancer patients, TIVAP-associated complications may occur and require early diagnosis and treatment. Since their invention four decades ago, complications have been identified and analyzed and practical guidelines created [4, 5, 6].

Initially, implantation technique and surgical complications were mostly in focus. Comparison of the two alternative approaches showed the success rate reported in different studies to be clearly in favor of the Seldinger technique, namely between 90 and 100% [7, 8, 9, 10, 11, 12], while the success rate for venous cutdown was only 70–94% [13, 14, 15, 16].

With further development of equipment and improvement of surgical technique, the most frequent complications changed to catheter-associated infections and thrombosis [17, 18]. The current literature reports complication rates between 6.9 and 17.7% [19, 20]. In the worst case, TIVAP-associated complications lead to TIVAP explantation. Fortunately, the TIVAP explantation rate described in recent studies is low with a few exceptions [21, 22]. Nevertheless, every single explantation means notable consequences for every patient, particularly a delay in ongoing chemotherapy for cancer treatment and difficulties for parenteral nutrition, resulting in increased morbidity, mortality, and costs [23, 24].

The aim of our study was to analyze complications under standardized operative and perioperative procedures and to identify risk factors for premature port catheter explantation.

## Material and methods

The retrospective cohort study was approved by Tuebingen University Ethics Committee (192/2018B02). The study cohort consisted of 1008 consecutive TIVAP implantations performed by one surgeon (G. M.) in patients aged 16 or older who received a TIVAP between January 1, 2016 and October 31, 2017 at the ambulatory operative center of the Department of General, Visceral, and Transplant Surgery, Tuebingen University Hospital, Germany. We retrospectively screened all patients treated with operation and procedure score OPS 5–399.5 (port implantation). Follow-up continued until the TIVAP was removed or the patient died. Follow-up time ended on October 31, 2018, so that patients were followed for minimum 1 year following implantation.

### Surgical procedure and standard anti-thrombosis prophylaxis

All operations were performed by the same high-volume general surgeon (G. M.) in local anesthesia using the well-established standardized open technique, i.e., cephalic vein cutdown [25].

Postoperatively patients were observed in the recovery room. After a chest X-ray was performed to confirm correct positioning of the TIVAP and to exclude pneumothorax, the patients were discharged.

A standard anti-thrombosis prophylaxis of low molecular weight heparin s.c., Fragmin P® 2.500 IU per day during the first 3 weeks after implantation, was recommended for all patients treated at our center. After every use and at least every 12 weeks, the system was flushed and blocked with 10–20 ml of NaCl 0.9%.

No perioperative antibiotic prophylaxis was given and immediate use of the TIVAP was allowed.

### Definitions of complications

Complications were defined as perioperative (during implantation and within 24 h after implantation), early (> 24 h and < 30 days after implantation), or delayed (more than 30 days after implantation). Classification of infections was performed according to the published guidelines [22]. Local infection was defined as redness, swelling, pain, and tenderness without signs of systemic infection. In cases involving fever and positive paired blood cultures from the central line and the peripheral vein with the same micro-organism, systemic inflammatory response syndrome/sepsis was diagnosed. Thrombosis was defined either clinically or radiologically (vascular sonogram or computed tomography (CT)). Thrombosis was differentiated as port chamber thrombosis, port tip thrombosis, or deep branch vein thrombosis. TIVAP-related complications consist of dysfunction, dislocation, disconnection, and fracture. Patient-related complications comprise hematoma, seroma, skin perforation, erythema, and pain without clinical signs of infection.

### Data collection and statistics

Data were collected with the hospital information database (SAP for Healthcare; SAP AG Walldorf). Collected data for each patient comprise demographic characteristics including age, gender, underlying disease, indication for permanent central venous catheter placement (chemotherapy, parenteral nutrition, combination), and follow-up time. In addition, the following surgical data were obtained: date of implantation, implantation side (right/left), operation time, implantation technique, and selected vein. Follow-up data consisted of complications (date, category, and treatment), indication, and date of TIVAP explantation. In the case of infection, pathogens were registered.

Mean values were compared with the Wilcoxon test (JMP® 14.0, SAS Institute, Cary, NC, USA). Complication rates were reported in absolute numbers, as rate per 1000 catheter days and percentage. Catheter survival was defined as the presence of the originally implanted catheter. Event was “TIVAP explantation due to complications”. Death with functioning TIVAP and planned explantation due to termination of therapy were considered censoring events. TIVAP survival curves were calculated with the Kaplan–Meier method and tested for significance using a log-rank test. A *p* value < 0.05 was considered significant. Results reported in the article, tables, and figures are reported as mean ± standard error of mean (SEM).

## Results

**Fig. 1:**
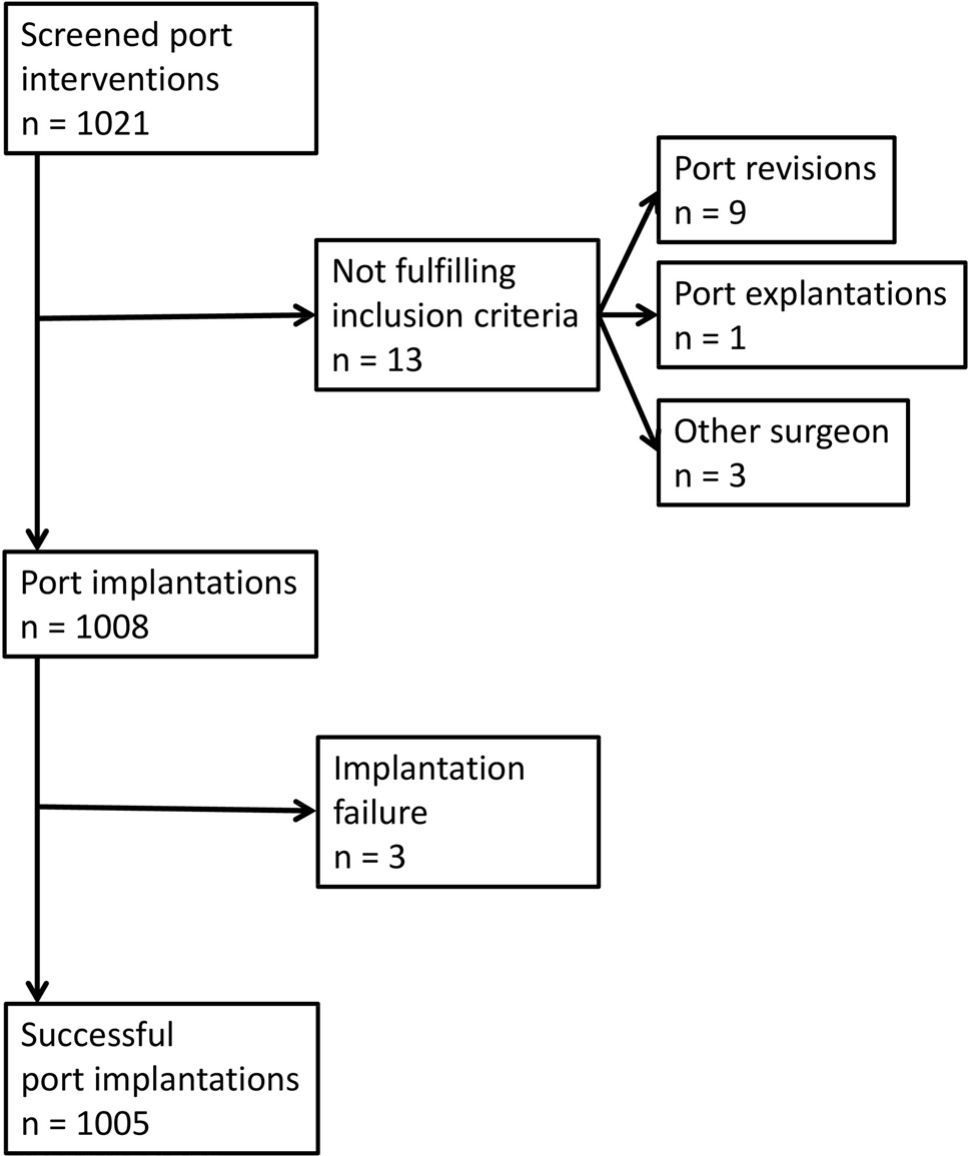
Flowchart showing the screening process

Altogether 1021 TIVAP procedures were screened (Fig. 1); 13 procedures had to be excluded because they did not meet the inclusion criteria. In nine cases, the OPS score 5–399.5 was erroneously used for TIVAP revision and in one case for TIVAP explantation. Three operations were performed by a different surgeon.

Successful surgical TIVAP implantation was achieved in 1005/1008 (99.7%) cases in 991 patients (14 patients received a second catheter). The following presented data refer to these 1005 successful implantations. Demographic and clinical characteristics are shown in Table 1. Mean age was 59 ± 0.5 (range 16–91) years, 742 (73.8%) patients were female, and 263 (26.2%) were male. The most prevalent underlying diseases requiring TIVAP implantation were breast carcinoma (432/1005, 43%), gastrointestinal carcinoma (252/1005, 25%), and gynecological tumors (142/1005, 14.1%).

**Table 1:** Demographic and clinical data

	Sample	Percentage
Successful TIVAP implantations	1005	100
Demographics		
Age, years (range)	59 ± 0.5 (16–91)	
Female/male	742/263	73.8/26.2
Underlying disease		
Breast carcinoma	432	43
Gastrointestinal carcinoma	252	25
Gynecological tumors	142	14.1
Leukemia and lymphoma	47	4.7
Head-neck tumors	24	2.4
Urological tumors	24	2.4
Bronchial carcinoma	23	2.3
Sarcoma	20	2
Other tumors	16	1.6
Dermatological malignancies	14	1.4
Benign diseases	11	1.1
Indication		
Chemotherapy	961	95.6
Chemotherapy and parenteral nutrition	21	2.1
Insufficient peripheral vein status	10	1
Parenteral nutrition	8	0.8
Withdrawal of blood	5	0.5

Values are reported as mean ± standard error of mean (SEM)

Most patients received a TIVAP for the administration of chemotherapy (961/1005, 95.6%), and 21 (2.1%) patients needed chemotherapy combined with parenteral nutrition.

Implantation side was mainly right (607 operations, 60.4%). The preferred blood vessel was the Vena cephalica in 958 (95.3%) cases. The Vena jugularis externa was used in 46 (4.6%) patients. In 10% of the implantations (also in the vein cutdown technique), a guidewire was additionally used to position the catheter tube.

Mean operation time was 30 ± 0.4 min (range 15–136 min). No intraoperative or perioperative complications occurred.

Overall, 120 (12%) complications were observed during the follow-up time of altogether 611.691 catheter days. Complications are summarized in Table 2. They comprised 32 (26.7%) early and 88 (73.3%) late complications. The explantation rate due to complications was similar for early (11/32, 34.4%) and late (34/88, 38.6%) complications. The most common complications were infections, which occurred in 47/1005 (4.7%) of the TIVAPs. Port catheter-induced blood stream infections were observed in 32 (3.2%; 0.052/1000 catheter days) cases and mainly occurred as a late complication 27/32 (84.4%). On average, blood stream infection happened after 179 ± 32 days. Conservative antibiotic therapy was successful in 11/32 (34.4%) cases, and 21/32 (65.6%) catheters had to be explanted due to systemic infection. Port pocket infections were reported in 15/1005 (1.5%; 0.024/1000 catheter days) TIVAPs, 5/15 (33%) were early complications, and 10/15 (67%) were late complications. On average, port pocket infection occurred after 166 ± 57 days. Therapy consisted of 12 (80%) explantations and antibiotic therapy in three cases (20%). With regard to disease, infections were mostly seen in patients with leukemia/lymphoma (5 infections/47 patients; 10.6%), followed by gastrointestinal cancer (22/252; 8.7%) and breast cancer (10/432; 2.3%). For a more detailed analysis of infections in the group of gastrointestinal cancer patients, a breakdown of diseases was performed: Infections were reported most frequently in gastric cancer (5/21; 23.8%), followed by pancreas carcinoma (9/56; 16.1%) and rectum carcinoma (1/33; 0.3%). Of the patients with rectum carcinoma, 17/33 (51.5%) had a diverting stoma. None of these patients suffered from an infection. Both local and systemic infections were mainly caused by *Staphylococcus epidermidis* (15; 32%), *Staphylococcus aureus* (12; 26%), and *Escherichia coli* (5; 11%). Temporal occurrence of pathogens shows differences: *Staphylococcus aureus* was mainly responsible for early infections, on average after 76 ± 33 days. *Staphylococcus epidermidis* and *E. coli* were found later after 264 ± 83 and after 382 ± 99 days (Wilcoxon test *p* = 0.0109 and *p* = 0.0302), respectively.

**Table 2:** Analysis of early, late and overall complications

Complication	Early complication > 24 h < 30 d 29,232 cd				Late complication > 30 d 582,459 cd				Overall complication 611,691 cd		
	*n*	%	/1000 cd	ex	*n*	%	/1000 cd	ex	*n*		/1000 cd
Infection (*n* = 47)											
Systemic	5	0.5	0.171	3	27	2.7	0.046	18	32		0.052
Local	5	0.5	0.171	5	10	1.0	0.017	7	15		0.024
Thrombosis (*n* = 36)											
Port chamber	-	-	-	-	3	0.3	0.005	-	3		0.005
Port tip	1	0.1	0.034	-	4	0.4	0.007	-	5		0.009
Deep branch vein	4	0.4	0.137	-	24	2.4	0.041	1	28		0.046
TIVAP-related complications (*n* = 20)											
Catheter dislocation	2	0.2	0.068	-	6	0.6	0.010	1		8	0.013
Fracture	3	0.3	0.103	2	2	0.2	0.003	2		5	0.008
Dysfunction	2	0.2	0.068	1	3	0.3	0.005	-		5	0.008
Extravasation	-	-	-	-	1	0.1	0.002	1		1	0.002
Port chamber dislocation	-	-	-	-	1	0.1	0.002	-		1	0.002
Patient-related complications (*n* = 17)											
Hematoma	5	0.5	0.171	-	-	-	-	-		5	0.008
Seroma	3	0.3	0.103	-	-	-	-	-		3	0.005
Skin perforation	-	-	-	-	4	0.4	0.007	4		4	0.007
Pain	2	0.2	0.068	-	1	0.1	0.002	-		3	0.005
Erythema	-	-	-	-	2	0.2	0.003	-		2	0.003
**Total**	32	3.2	1.094	11	88	8.8	0.15	34		120	0.196

*cd* catheter days; *ex* explantation

The second most common complication was thrombosis, which was evident in 36/1005 (3.6%) cases, corresponding to 0.06/1000 catheter days. Of the thromboses, 5/36 (13.9%) were early complications and 2/36 occurred during the first 3-week phase of recommended anticoagulation, and 31/36 (86.1%) were late complications. Average time of occurrence was 192 ± 35 days. Localizations of the thrombus were the port chamber in three (8.3%) cases, port tip in five (13.9%), and deep branch vein in 28 (77.8%) cases. Nearly half of the patients (17/36; 47.2%) were asymptomatic and thrombosis was diagnosed as an incidental finding in staging computed tomography. In two patients (5.6%), thrombosis resulted in segmental lung embolism. In 35/36 (92.2%) thromboses, conservative anticoagulative therapy was successful. In one of the two patients with a pulmonary embolism, the TIVAP had to be removed because the patient was already anticoagulated when the lung embolism occurred.

In view of underlying disease, thrombosis was found mainly in the group of leukemia/lymphoma patients, namely in 2/47 (4.3%), followed by breast carcinoma 18/435 (4.1%) and gastrointestinal tumor 9/252 (3.6%) patients.

TIVAP-related complications were documented in 20/1005 (2%) cases, corresponding to 0.033/1000 catheter days; 7/20 (35%) were classified as early and 13/20 (65%) as late complications. Most frequently dislocation of the catheter was seen in 8/20 (40%), followed by fracture in 5/20 (25%) cases and dysfunction also in five cases. Therapy consisted of explantation (7/20, 35%), revision (7/20, 35%), and conservative therapy (6/20, 30%).

Patient-related complications occurred in 17/1005 (1.7%) patients, corresponding to 0.028/1000 catheter days. The majority of the complications occurred early (10/17, 58.8%), namely hematoma and seroma. No pneumothoraces were detected. Skin perforation was seen clearly later (131–337 days after implantation) in four patients and resulted in explantation in all cases. Of the patient-related complications, 12/17 were treated conservatively with local therapy and analgesia.

**Fig. 2:**
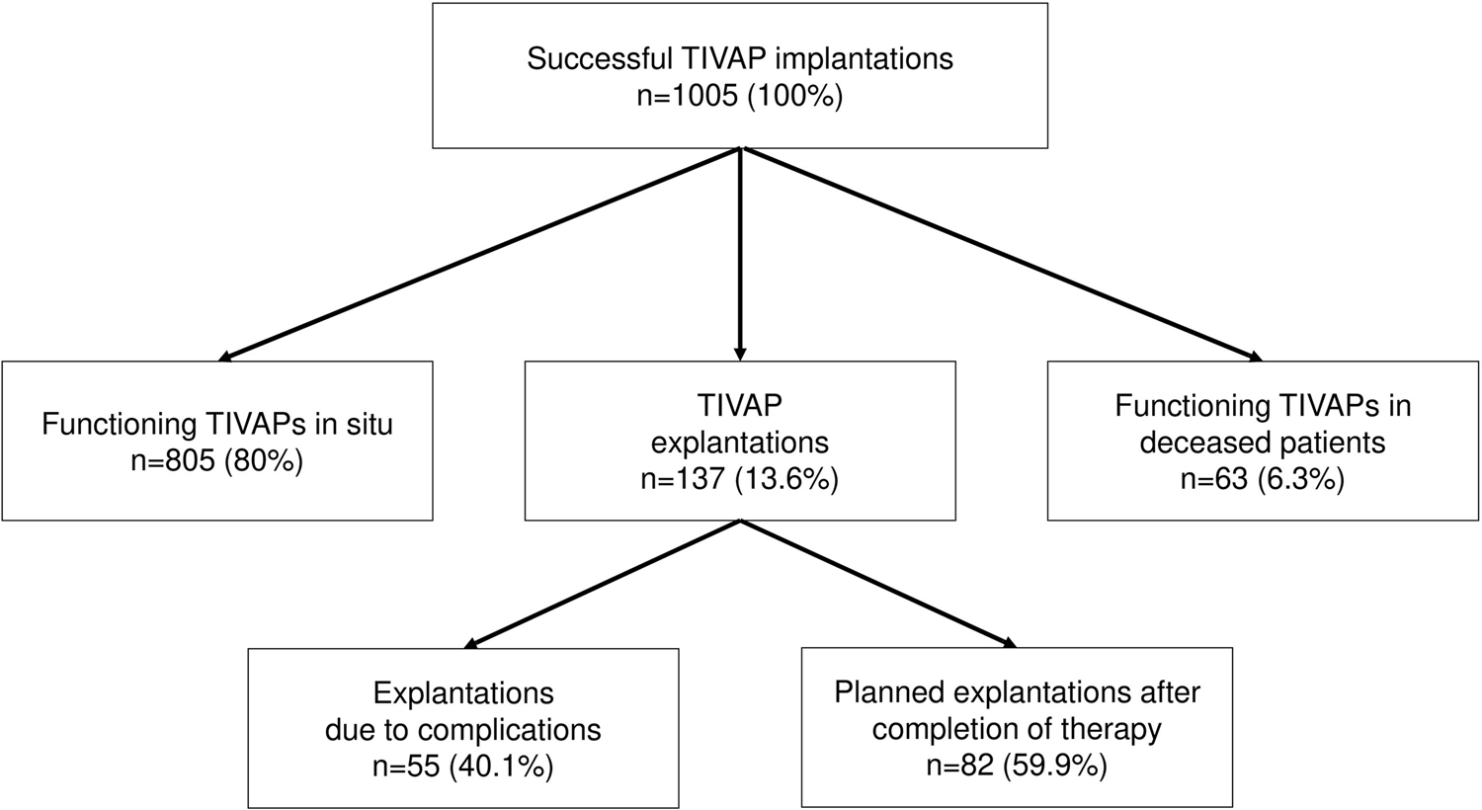
Follow-up flowchart

At the end of the observation period, 805/1005 (80%) of the implanted TIVAPs were functioning in situ (Fig. 2).

During the study period, 63 patients died with a functioning TIVAP. Of 137 TIVAP explantations, 82 (59.9%) were according to plan after completion of the therapy regimen, while 55 (40.1%) TIVAPs had to be removed due to complications. Explantation due to complications was indicated after approximately 179 ± 25 days and therefore TIVAP in situ time was significantly shorter than for planned explantations after 379 ± 20 days (*p* < 0.0001).

**Fig. 3:**
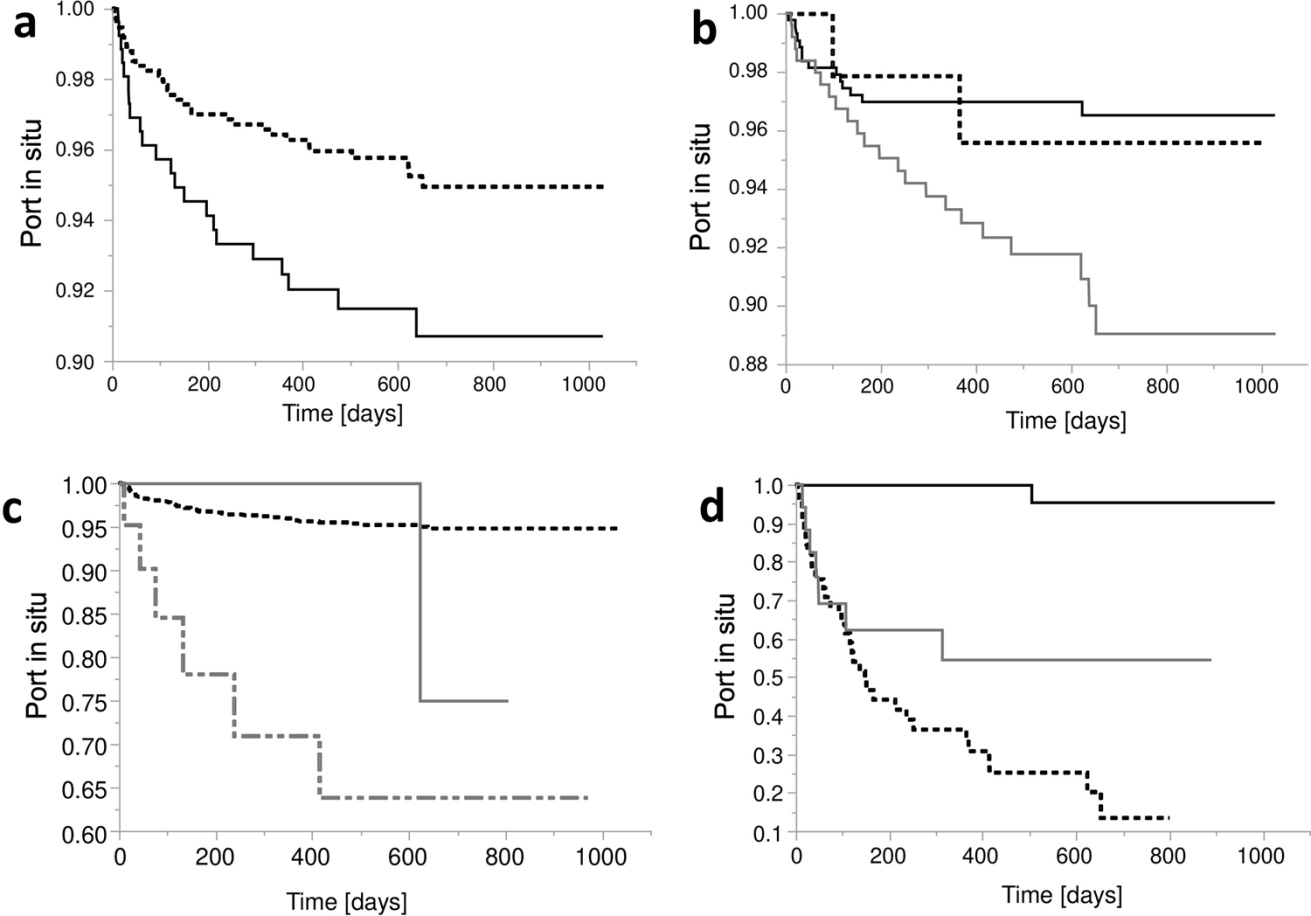
Kaplan–Meier curves for catheter survival. **a**) relevant factor: **gender;** female: dotted line, male: black line, * *p* = 0.01. **b**) relevant factor: **disease;** breast cancer: black line, gastrointestinal cancer: grey line, leukemia/lymphoma: dotted line, * *p* = 0.004 breast cancer vs. gastrointestinal cancer. **c**) relevant factor: **indication;** chemotherapy: dotted black line, parenteral nutrition: grey line, combination of chemotherapy and parenteral nutrition: dotted and dashed grey line, * *p* < 0.001 chemotherapy vs. chemotherapy and parenteral nutrition. **d**) relevant factor: **type of complication;** thrombosis: black line, TIVAP-related complication: grey line, infection: dotted black line, * *p* < 0.0001 thrombosis vs. TIVAP-related complication, * *p* < 0.0001 thrombosis vs. infection

Kaplan–Meier curves for TIVAP survival in relation to the event “explantation due to complication” are shown in Fig. 3. Parameters that correlated with TIVAP explantation were gender (Fig. 3a) (port in situ: female 95% vs. male 91%, *p* = 0.01), underlying disease (Fig. 3b) (breast carcinoma 97% vs. gastrointestinal 89%, *p* = 0.004), indication (Fig. 3c) (chemotherapy 95% vs. combination of chemotherapy and parenteral nutrition 64%, *p* < 0.0001), and type of complication (Fig. 3d) (infection 13.4% vs. TIVAP-related complication 54% and thrombosis 95%, *p* < 0.0001). In contrast, catheter survival was not affected by implantation side, operation time less or more than 30 min, with or without the need for a guidewire, or by patient age.

## Discussion

In the present study, more than 1000 TIVAP implantations were analyzed in order to identify risk factors for premature catheter explantation. The surgical success rate of 99.7% in our series and the large cohort provide an excellent foundation for further analysis. The extremely low failure rate, the absence of intraoperative and perioperative complications, and the consistently short operating time of 30 ± 0.4 min in our study might be caused by the fact that all operations were performed by the same highly experienced surgeon.

The overall complication rate in our study was low at 0.196 complications per 1000 days or 12% altogether. In accordance with other studies, the temporal distribution was in favor of late complications (8.8%) as compared to early complications (3.2%) [17, 19]. From this, it follows that only 5.5% of the implanted TIVAPs had to be explanted due to complications. The range in other series varies between 4.3 and 29.8% [20, 21, 22, 26]. The high explantation rate of 29.8% in the study by Kilic et al. might be explained by their high infection rate of 16.1% [26]. In our study, infections occurred in 4.7% of TIVAPs and are the most common complication as well as the most common reason for TIVAP explantation. It must be underscored that TIVAPs had to be explanted in only two-thirds of patients with a systemic infection as compared to clearly higher explantation rates reported by Vida et al. (81%), Ahn et al. (88%), and Teichgräber et al. (100%) [17, 27, 28]. The importance of early diagnosis and therapy of infections was proven by Mermel et al. [29]. For TIVAP implantation in our hospital, no perioperative antibiotic is administered and the applicability of this procedure was confirmed by our study data with overall only five local and five systemic early infections. Infection rates in recent studies vary between 1.6 and 50% [17, 19, 20, 30, 31]. The lowest infection rate was found by Ma et al. in a study cohort consisting of only breast cancer patients [19], and this aspect was confirmed in the present data showing a low infection rate of 2.3% for this patient group. The highest infection rate was found by Viana Taveira in pediatric patients, and nearly 70% of whom had lymphoma/leukemia [31]. In accordance with these data, the small group of patients with lymphoma/leukemia in our study showed the highest infection rate, namely 10.6%. Increased infection rates in hemato-oncology malignancies were reported in several studies and intensive chemotherapy and impairment of the immune system were seen as a causal relationship [28, 30, 32]. Zerati et al. and Shim et al. justify their increased infection rate with the high rate (20.5%) of stationary patients [22, 30]. In our study, all operations were performed on an out-patient basis, which might be beneficial for a lower infection rate.

In the present study, the infection rate for patients with gastrointestinal cancer was 8.7% and was reported most frequently in patients with gastric cancer (23.8%), followed by pancreas carcinoma (16.1%) and rectal carcinoma (0.3%). Half of the patients with rectal carcinoma in our study had a diverting stoma, but none of these patients came down with an infection.

Of the infections, 79% were caused by gram-positive pathogens, mainly *Staphylococcus epidermidis* and *Staphylococcus aureus*, which is consistent with the findings of other current studies [27, 31, 33]. A shift toward gram-positive bacteremia in cancer patients was observed many decades ago and not only the use of antibiotic prophylaxis but also the existence of an indwelling catheter and the nature of chemotherapy are held responsible for this [34, 35].

The second most common complication observed in our study was thrombosis in 3.6% of TIVAPs. Standard anti-thrombosis prophylactic regimen in our center consists of the recommendation to administer low molecular weight heparin during the first 3 weeks after implantation and to flush and block the system with 10–20 ml of NaCl 0.9% after every use and at least every 12 weeks. Two thromboses occurred during the first 3-week phase of recommended anticoagulation. Since this is a retrospective study, it is unknown if the patient followed the recommendation. The primary prevention of TIVAP-associated thrombosis with anticoagulant drugs is currently not recommended by most scientific societies and guidelines [36, 37]. Instead, risk stratification for previous thrombosis, type, location, and stage of cancer and hereditary defects is suggested [38]. It is debatable whether the recommendation for anti-thrombosis prophylaxis at our center is too general and some patients received it unnecessarily. The results of our study show that the standard anti-thrombosis prophylactic regimen at our center needs to be discussed because it appears to provide little benefit and therefore could be changed to a risk-stratified regimen.

Nearly half (47.2%) of the patients were asymptomatic and thrombosis was diagnosed as an incidental finding in staging computed tomography. Asymptomatic thrombosis was also recorded in prospective studies and incidences of 71% and 94% were reported (21, 68). In only one case of thrombosis resulting in a segmental lung embolism during anticoagulative therapy did the TIVAP have to be removed. All other thromboses were treated successfully with anticoagulative therapy.

In three cases, thrombi were localized in the port chamber, which in the proper meaning of the word is not really a thrombosis, but more probably a consequence of poor nursing practices with insufficient push-pause cleaning of the line after blood sampling.

Although TIVAP-related complications occurred very rarely, namely in only 2% of TIVAPs, their occurrence significantly affected catheter survival. In contrast, all patient-related complications were able to be treated conservatively with the exception of skin perforation in four cases when a TIVAP had to be explanted.

## Conclusion

In conclusion, our large single-center series shows that standardized operative and perioperative procedures for TIVAP implantation provide excellent results and a low explantation rate. Risk factors for unplanned explantation were gender, underlying disease, indication, and kind of complication.
